# Bioenergetic impairment in Gulf War illness assessed via ^31^P-MRS

**DOI:** 10.1038/s41598-024-57725-4

**Published:** 2024-03-28

**Authors:** Beatrice Alexandra Golomb, Jun Hee Han, Alexander Fung, Brinton Keith Berg, Bruce J. Miller, Gavin Hamilton

**Affiliations:** 1 Department of Medicine, University of California, San Diego, 9500 Gilman Drive # 0995, La Jolla, CA 92093-0995 USA; 2 Department of Radiology, University of California, San Diego, La Jolla, CA 92093 USA; 3 Present Address: Clement Park Dental Care, Littleton, CO 80123 USA

**Keywords:** Gulf War illness, Mitochondrial, ^31^P-MRS, Phosphocreatine, Bioenergetics, Gender differences, Muscle weakness, Muscle, Mitochondria

## Abstract

Time for post-exercise phosphocreatine-recovery (**PCr-R**), deemed a robust index of mitochondrial function in vivo, was previously reported to be elevated (signifying impaired ATP production) in veterans with Gulf War illness (**GWI**). Here we sought to replicate the finding and assess the impact of contravening previous eligibility requirements. The replication sample comprised white males. Cases reported ≥ moderate muscle-weakness to match the organ assessed to an organ affected; controls lacked recent headache or multiple symptoms. The expansion sample added cases without muscle-weakness, controls with recent headache, females, nonwhites. PCr-R, following pedal-depression-exercise, was compared in veterans with GWI versus controls (sample N = 38). In the replication sample, PCr-R results closely matched the prior report: PCr-R veterans with GWI mean(SD) = 47.7(16.5); control mean(SD) = 30.3(9.2), p = 0.017. (Prior-study PCr-R veterans with GWI mean(SD) = 46.1(17.9), control mean(SD) = 29.0(8.7), p = 0.023. Combined replication + prior samples: p = 0.001.) No case–control difference was observed in the expansion sample. In cases, PCr-R related to muscle-weakness: PCr-R = 29.9(7.1), 38.2(8.9), 47.8(15.2) for muscle-weakness rated none/low, intermediate, and high respectively (p for trend = 0.02), validating desirability of matching tissue assessed to tissue affected. In controls, headache/multiple symptoms, sex, and ethnicity each mattered (affecting PCr-R significantly). This study affirms mitochondrial/bioenergetic impairment in veterans with GWI. The importance of careful case/control selection is underscored.

## Introduction

Gulf War illness (**GWI**) is an exposure-induced chronic multi-symptom health problem reported to affect on the order of a third of the ~ 700,000 U.S. veterans deployed to the 1990–1991 Operations Desert Shield and Desert Storm (Persian Gulf War). Deployed veterans from other nations were also affected^1–4^. The ground war lasted only four days and many of those deployed never saw combat. Combat stress (although it shows a dose-dependent relationship to post-traumatic stress disorder, a different condition) is not an independent predictor of GWI^5^.

GWI is tied to drug/chemical/environmental exposures which occurred in unprecedented number, variety and intensity, with many new, unique and excessive exposures. Particular attention has gone to carbamate and organophosphate acetylcholinesterase inhibitors^6^ which have been shown to exert toxicity through impaired mitochondrial function and oxidative stress^7–9^.

Mitochondrial impairment has emerged as one candidate mechanism for GWI^10–19^ that could account for the protean nature of symptoms, varying from person to person, with an emphasis on fatigue, brain and muscle problems—i.e. post-mitotic tissues with high energy demand—and the variable latency in onset of symptoms^20–23^.

It was previously reported that veterans with GWI had impaired mitochondrial function assessed by the time constant of phosphocreatine (**PCr**) recovery after exercise, determined by ^31^phosphorus-magnetic resonance spectroscopy (^**31**^**P-MRS**)—a technique that procures spectra of phosphorus-containing products^10^.

PCr, a backup energy source to muscle, is depleted with exercise. Recovery rate is dependent on the rate of ATP production, so a prolonged recovery time constant (**PCr-R**), signifying impaired recovery kinetics, is viewed as a robust in vivo index of mitochondrial function^24^. We refer to “mitochondrial/bioenergetic” impairment to acknowledge the fact that, although prolonged PCr-R has been characterized in this way^24^, in fact mitochondrial respiratory chain function *per se* is not the sole determinant of the rate of the ATP synthesis that underlies the recovery time constant—this can be construed as an index of “mitochondrial” function quite broadly defined (i.e. mitochondrial ATP production), or alternatively as an index of bioenergetic function. Other factors potentially including substrate access and rate of glycolysis^25^ could also serve as contributors. However, recent muscle biopsy data, that affirm a relation between mitochondrial impairment and GWI severity, suggest that mitochondrial impairment is at least an important factor for veterans with GWI^26^.

In that study, cases and controls were carefully selected. Because mitochondrial impairment can affect different tissues to different extents, it is recognized that in assessing for mitochondrial impairment, it is desirable to sample affected tissues or organs^27–30^. Conversely, in a study sampling a specific organ, it is desirable to focus on individuals in whom that organ is affected. In the previous study, all veterans with GWI had at least moderate self-rated muscle weakness. The control group did not include individuals with recent headache; headache has been tied to mitochondrial impairment^31–33^.

We sought to reassess PCr-R in veterans with GWI versus controls, for the aim of both replicating the prior finding and also assessing whether similar findings are seen if participant selection criteria are materially relaxed; each of these constitute original contributions to the literature. Specifically, we sought to encompass veterans with GWI that did not report muscle weakness, and controls who, although failing to meet CDC or Kansas symptom-inclusion criteria for chronic multi-symptom illness, nonetheless reported recent headache and/or recent multiple symptoms. Additionally, we sought to broaden the sample to both sexes and varied ethnicities, recognizing that this may increase variance or alter findings.

## Methods

### Ethics statement

The study protocol was approved by the University of California, San Diego, Human Research Protections Program (Project #130577) and the Department of Defense Human Research Protection Office (Award #W81XWH-13-1-0232). All participants gave written informed consent to participate. All methods were performed in accordance with relevant guidelines and regulations.

### Design

Case–control study.

### Participants

Veterans with GWI cases comprised 20 veterans deployed to the Gulf theater of operations between August 1, 1990 and July 31, 1991, who met both CDC^34^ and Kansas^35^ symptom inclusion criteria for GWI. CDC criteria require at least one chronic symptom, present for at least six months, beginning during or after Gulf participation, in at least two of the three domains of fatigue, mood-cognitive and musculoskeletal. Kansas criteria also consider symptoms persistent for at least six months, with onset during or after Gulf participation, but require qualifying symptoms in at least three of six domains of fatigue-sleep, pain, neurological-mood-cognitive, gastrointestinal, respiratory, and dermatologic. For a domain to qualify, the symptoms within the domain must be either multiple (> 1) and/or at least moderate in severity—i.e. more than mild. Conditions like lupus or multiple sclerosis that could produce similar symptoms for reasons other than GWI were exclusionary.

Controls were nonveterans, could not meet either CDC or Kansas symptom inclusion criteria, and could not meet Kansas exclusion criteria—they could not have a condition such as lupus or multiple sclerosis that could lead to similar symptoms (even if such symptoms were not present).

As this study seeks to reassess PCr-R in veterans with GWI vs controls in an independent sample, participants in the previous ^31^P-MRS study^10^ were ineligible to participate in the present study.

### Recruitment

Gulf War veterans and controls were recruited via outreach to existing rosters of Gulf War veterans who had participated in or inquired about Gulf War studies (excluding those who had participated in the bioenergetic study), supplemented by social media outreach through Gulf War veteran groups, and “friends and family” outreach by study participants. Additionally, ResearchMatch was used to recruit, particularly for healthy controls.

### Metal screening

Prospective participants were screened prior to enrollment and again on the day of their visit to ensure ^31^P-MRS eligibility which required absence of shrapnel, no prosthetic devices or other metal that might pose problems with magnetic resonance assessment; and weight ≤ 300 pounds. Those who had engaged in welding as a hobby but wished to participate received an orbital x-ray first to ensure absence of metal fragments in the eye.

### ^31^P-MRS

An abridged version of the methods is provided from the prior paper^10^, which can be viewed for full methodological details. The following sentences are quoted from the previous methods, but ellipses between quoted elements have been removed for ease of viewing.

“^31^P-MR spectra were acquired on a 3 Tesla GE Signa EXCITE HD scanner (GE Healthcare, Waukesha, WI). Participants were scanned in the supine position. The ^31^P-MR spectra were collected with a 5-inch diameter surface coil, using a slice selective free induction decay sequence with a repetition time of 3 s. Spectra had a sampling interval of 0.2 ms. For muscle spectra, the coil was placed under the calf. For the exercise protocol, a spectrum was collected every 3 s during 2 min of rest (providing resting muscle spectra), then 5 min of exercise (repetitively depressing, as far as they were able, a metal-free pedal, similar to depressing a car pedal, with elastic bands providing resistance). This was followed by 6 minutes of recovery. A velcro strap across subjects' thighs limited extraneous movement during exercise.”

All ^31^P-MR spectra were acquired with 5-inch outer diameter surface coil. The main muscle localization would be a function of coil position, with the signal being strongest close to the coil and decreasing with distance. The slab volume was positioned to minimize contributions from the skin with the slab thickness set to the maximum available value, to maximize signal. The volume of muscle providing signal is primarily determined by the coil response profile—as a rule of thumb, the penetration depth of a loop coil is approximately equal to its inner diameter.

### ^31^P-MRS analysis

Full details of ^31^P-MRS analysis are available elsewhere^10^. As above, the following sentences are quoted from the previous methods, but ellipses between quoted elements have been removed for ease of viewing.

“The inherently low signal-to-noise of ^31^P-MR spectra and the complex overlapping peak structure of the spectra were addressed via AMARES algorithm^36^ included in the MRUI software program^37^, using prior knowledge adapted from an approach previously used by Dr. Hamilton at 1.5 T^38^.” The pH was calculated at two points; pre-exercise at rest (sum of spectra acquired in the 2 min before the start of exercise) and post-exercise (sum of spectra acquired between 1- and 3-min post exercise). The post-exercise PCr-R time constant was procured, as relative change in the area. Intracellular pH values were determined by manually estimating the Pi-PCr chemical shift for both the resting and post-exercise spectra, as previously described: This method was found to produce the most consistent results, and there is reportedly no benefit in using more complex approaches^39,40^.

PCr-R was procured by fitting an exponential to the PCr recovery curve, with PCr-R defined as tau, the time constant of the exponential, measured in seconds. Tau is determined by the rate of oxidative ATP synthesis, independent of effort^24^. As above, we analyzed the spectra, summing the spectra acquired between 1 and 3 min post the end of exercise and estimated the pH from the PCr-Pi chemical shift. We compared this to the resting muscle pH which was calculated from the PCr-Pi chemical shift in summed spectra acquired before the start of exercise. Assessments were conducted blinded to case–control status, and passed for data entry after all veterans with GWI and controls had completed participation.

### GWI symptoms

In addition to Kansas and CDC screening surveys, participants completed the UCSD GWI Symptom Survey, in which they rated presence and severity in the prior two weeks for each of 20 symptoms. Component symptoms had been determined in a previous GWI study, with symptoms selected for presence in the previous two weeks in at least 50% of veterans with GWI in that study^11^. The UCSD GWI symptom score, summing 0–10 ratings across the 20 symptoms, has been validated against Kansas GWI criteria, objectively measured physical function, and in vivo assessments of mitochondrial function (data available on request).

Rated items included: aches and pains, joint pain, tiredness, sleep problems, having to go back and recheck things, muscle pain, trouble recalling words or names, irritable, impatient, attention problems, too little energy to get up and do things, headache, anxiety, muscle fatigue, fatigue with exertion, tinnitus, forgetting where going/what doing, dry skin, cold hands or feet, and reading comprehension problems.

Self-rated muscle weakness had been previously validated in veterans with GWI against objectively measured muscle function via the validated lower extremity Summary Performance Score^41,42^, to which it significantly related: r = − 0.47, p = 0.001, averaging duplicate measurements of each procured ~ one week apart.

### Power, sample size, effect size

The overall sample size calculation supposed 20 per group, with matched pairs (providing for dependent assessment). The matched pairs did not consider the expansion criteria in matching.

The smaller replication sample would lose the matching property and under those original assumptions, there was 80% power, with two-tailed alpha of 0.05, to detect an effect size of 0.45 standard deviations—that is to say, excellent power, to detect even a relatively modest sized effect. (The same sample size, in the event of loss of dependence (pair matching), provides 80% power (two-sided alpha of 0.05) only if the effect size is large—0.91 SD.) However, the previous replication sample demonstrated a large effect size, and showed significance with an N of 14 (seven per group). Based on this, it was anticipated that even if expanding the sample beyond original criteria did not sustain the observed effect, the replication sample would have good prospects to have adequate power provided the replication sample group was of at least comparable size.

Power calculations were conducted using G*Power 3.1.9.7^43^. The t-test family of tests were employed, using the “sensitivity” test for two independent samples to determine effect size required to achieve the designated power. (The original sample size calculation using the paired values employed the same software, but the “a priori” test with dependent values t-test, to determine the desired sample size.)

### Statistical analyses

Descriptive analyses were conducted to evaluate participant characteristics in the replication sample as well as the expansion sample, considering participants with successful PCr-R procurement. Participants with missing data—inability to secure a PCr-R time constant—were omitted from analysis; data were not imputed for these individuals. Descriptive analyses comprised means and standard deviations for continuous variables and percentages for categorical variables. PCr-R was compared in cases versus controls via t-tests of difference in mean values, for the replication sample and the expansion sample. Findings for the replication sample were compared to the findings from the sample it seeks to replicate. A merged analysis—functionally the first stage of cumulative meta-analysis—was conducted, combining individual-level data from the replication sample and from the original published sample, and testing case–control differences in PCr-R. Recall, no participants were in common between the two studies.

The relations of PCr-R to those participant characteristics that differed in the replication versus expansion sample were examined. Thus, the relation of PCr-R to muscle weakness (cases), to sex and ethnicity, and to headache status and multiple symptoms (controls) were assessed. For muscle weakness, in addition to the 2-stratum comparison via t-test, a 3-stratum assessment was performed, using the nonparametric trend test developed by Cuzick^44^.

Analyses used Stata^®^ (College Station, TX) versions 8.0 and 12.0. 2-sided p-values < 0.05 were deemed to designate statistical significance.

## Results

### pH values

No significant difference was found between the pre-exercise and post-exercise pH values. This was true in the total sample, and in cases and controls assessed separately.

### PCr-R procurement

**Figure 1:**
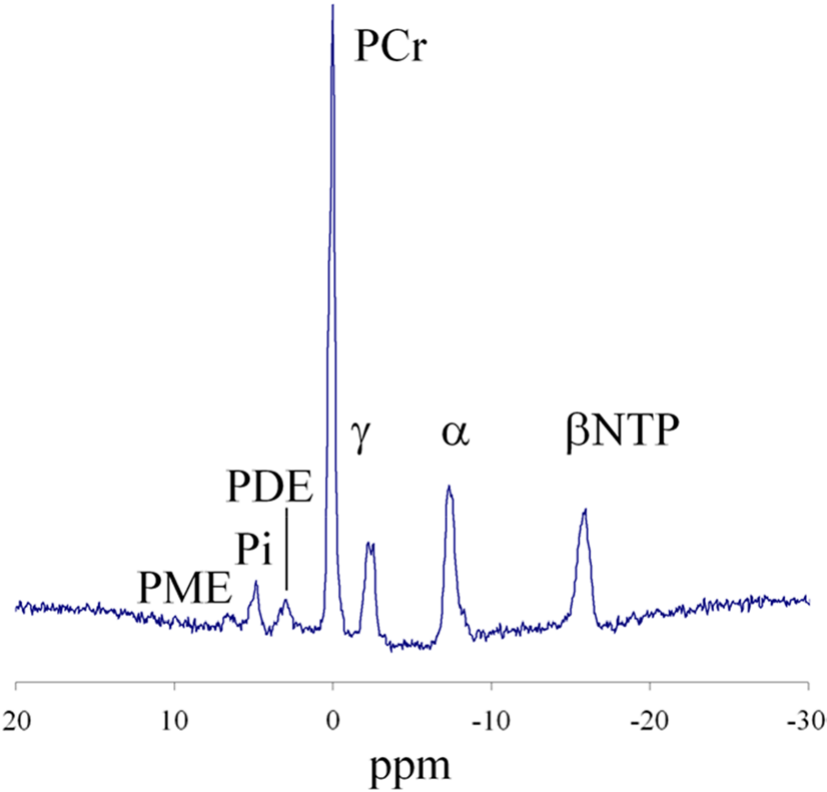
^31^P-MRS spectrum from resting muscle.

Figure 1 shows an example of a muscle spectrum at rest, in which the PCr peak can be seen.

**Figure 2:**
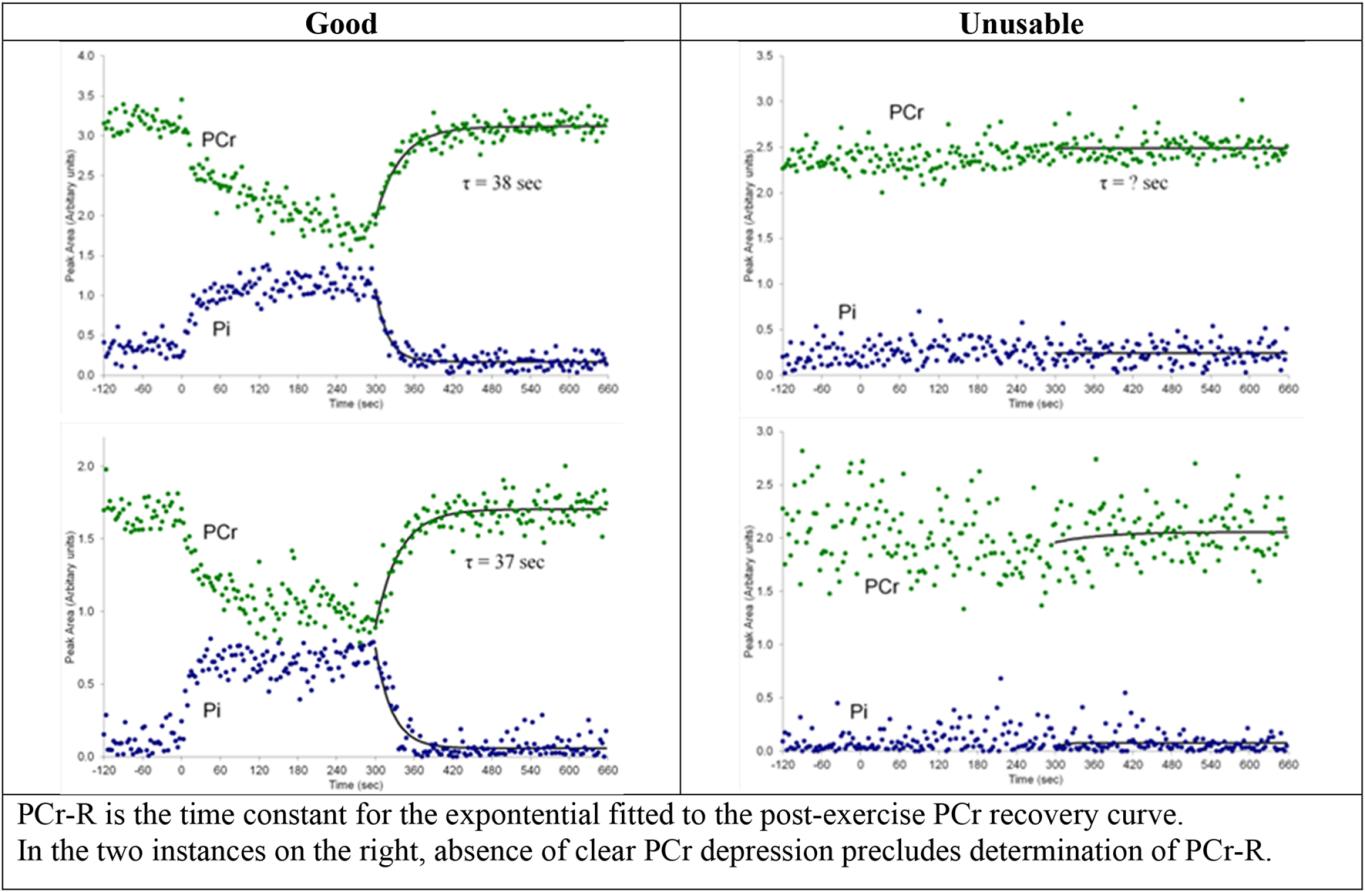
Muscle PCr tracked with rest, exercise and recovery.

Figure 2 illustrates successful and unsuccessful exercise efforts, the former displaying PCr depression with exercise permitting the fitting of an exponential to the PCr recovery curve. Where PCr is successfully depressed with exercise, the time constant of the recovery curve exponential equates to PCr-R. Where no clear depression is observed and no exponential can be fitted, no PCr-R can be ascertained. PCr-R was successfully ascertained in 38 participants, 20 veterans with GWI and 18 controls. Since pH did not significantly differ in the post-exercise relative to the pre-exercise phase, this enables PCr-R to serve as a proxy for ATP production.

### Participant characteristics

Table 1 shows participant characteristics. Participants were mostly male and white, consistent with the demographics of veterans with GWI. Veterans with GWI, by selection, had materially higher numbers of affected Kansas domains, and higher summed Kansas scores. They had higher UCSD GWI symptom scores, on which selection did not occur—reflecting recent symptoms rather than chronic symptoms. (Because data were not passed for analysis until conclusion of the study, we had no foreknowledge that two controls did not successfully depress PCr-R. This led to modification of statistical power, with 20 vs. 18 in unpaired analysis, modestly affecting the power calculation, relative to the 20 per sample unpaired analysis in the methods. Considering the effect size for which 20 per sample would have 80% power, the power would now be 78%.)

**Table 1:** Participant Characteristics.

Characteristics	Replication n = 16		Expansion n = 38	
	Veterans with GWI	Controls	Veterans with GWI	Controls
Age (years)	53.9 (6.1)	54.4 (6.7)	53.2 (6.1)	54.5 (6.8)
% Male	100	100	90.0	88.9
% White	100	100	75.0	83.3
% Married	100	77.8	85.0	50.0
# of Kansas Domains	5.29 (0.76)	0 (0.0)	4.75 (1.1)	0 (0.0)
Kansas score	49.0 (14.0)	0 (0.0)	41.3 (16.7)	0 (0.0)
% ≥ Moderate Weakness	100	0	65.0	0
% Headache (last 2 weeks)	100	0	85.0	27.8
UCSD GWI symptom score	127 (18.0)	1.80 (3.61)	104 (38.8)	5.91 (9.8)

^31^PMRS results were passed from the MR physicist for data entry after conclusion of all participant visits to enhance data integrity. In the few instances in PCr-R could not be ascertained, this was only made known a posteriori.

20 veterans with GWI and 18 controls underwent ^31^P-MRS and successfully engaged in test muscle exercise that depressed PCr, enabling PCr-R to be determined. Instances of unsuccessful PCr-R led to case–control imbalance in ethnicity. Balance was enabled for the replication sample by focusing on white participants.

### PCr-R

Table 2 shows results of PCr-R in the replication sample, and in the published sample, which it seeks to replicate. Results are strikingly similar, with PCr-R one second longer in cases as well as in controls in the replication sample. (Veterans with GWI and corresponding controls are now several years older than at the time of the prior study.) For each sample, PCr-R was ~ 50% greater (prolonged recovery time) in veterans with GWI cases than in controls. Findings were significant for the independent replication sample, as they had been for the previous published sample. In analysis of the combined sample, merging participants from the two groups, the significance of the difference is striking (p = 0.001).

**Table 2:** PCr-R is Prolonged in Veterans with GWI: Independent Replication Sample Coheres with Prior Published Finding.

Control	Case	P
Replication sample (n = 16)		
30.3 (9.2)	47.7 (16.5)	0.017
Previous finding^10^ (n = 14)		
29.0 (8.7)	46.1 (18.0)	0.023
Combined sample (n = 30)		
29.7 (8.7)	46.9 (16.6)	0.001

Values under the control and case columns are PCr-R mean (SD). PCr-R is measured in seconds.

PCr-R did not differ, as effect or trend, for veterans with GWI cases versus controls in the expansion sample (p = 0.72). The dissociation from findings in the replication sample justifies the examination of the relation of PCr-R to the features that distinguish the replication from the expansion sample.

Table 3 examines how muscle weakness self-rating related to PCr-R in cases. No control cited muscle weakness, which therefore cannot be evaluated separately in this group. Consistent with the importance of matching the organ assessed to an organ that is affected in assessments for mitochondrial impairments, here we see a graded relationship between muscle weakness and prolonged PCr-R in veterans with GWI. Those with no or minimal muscle weakness, although they are symptomatic in other ways and *may presumably* have mitochondrial impairment in other tissues, show no evidence of mitochondrial impairment when muscle is assessed via PCr-R. Regression analysis with robust standard errors predicting muscle weakness by PCr-R, in veterans with GWI, shows a $\beta$(SE) of 0.086(0.036), p = 0.028.

**Table 3:** PCr-R in GWI Cases, Stratified by Muscle Weakness.

	Weakness rated 0–5	Weakness rated 6–10	
	N = 7	N = 13	
Two group stratification			
PCr-R, Mean (SD)	31.1 (6.95)	45.4 (13.0)	
P for difference	0.01		

	None/Low weakness (rated 0–4)	Intermediate weakness (rated 5–7)	High weakness (rated 8–10)
	N = 4	N = 8	N = 8
Three group stratification			
PCr-R, Mean (SD)	29.9 (7.1)	38.2 (8.9)	47.8 (15.2)
P*	0.02		

*Assessment of significance for the three group stratification was performed using nonparametric test of trend (“nptrend” in Stata^®^).

Of note, while nonwhite and nonmale *cases* did not differ significantly from white male cases in their PCr-R values, nonwhite and/or nonmale *controls* did differ significantly from white male controls, with materially longer PCr-R (p = 0.033). PCr-R was also strongly and significantly tied to recent headache and recent multiple symptoms in controls. (Because this is of no interest to a non-Gulf War audience, these findings will be published separately.) These findings reinforce the importance of careful case–control selection, the potential disparate findings by sex and ethnicity and the possible need to consider symptoms that are not “chronic” in defining healthy controls.

## Discussion

This study provides several original findings. For the first time, it replicates evidence of bioenergetic impairment via post-exercise PCr recovery time constant, in a sample selected for similarity to the previous sample in which this was shown. That is, our findings in a "replication" sample duplicate the previous report of impaired bioenergetics in veterans with GWI compared to ethnically matched controls in a homogeneous male sample. PCr-R—the post-exercise time constant of PCr recovery assessed on ^31^P-MRS—was significantly prolonged in veterans with GWI relative to healthy controls, heeding appropriate case–control selection. For the first time, it initiates first stage of a cumulative meta-analysis, revealing very strong significance where the selection characteristics are retained. For the first time, it shows that expanding beyond these careful selection criteria compromises ability to demonstrate this effect (e.g. including controls with headache or GWI cases without muscle weakness). For the first time, it shows a significant relationship between degree of self-rated muscle weakness and severity of PCr-R prolongation.

To review criteria for the replication sample, veterans with GWI (case participants) were chosen to be symptomatic in the assessed organ. Controls were chosen to be without recent headache, or multiple symptoms, in recognition of the documented relation of headache, as well as multiple symptoms, to mitochondrial impairment^31–33^. Our findings also substantiate the apparent importance of each of these selection choices. The strong relation within veterans with GWI of muscle weakness to PCr-R underscores the potential importance of matching the assessed organ to those who are symptomatic in that organ when assessing mitochondrial status^28^. It adds further objective validation, incidentally, to single-item self-ratings of muscle weakness, which here related significantly to PCr-R and which have also been validated against objectively measured physical function in veterans with GWI.

Conversely, our findings underscore the importance, with controls who do not meet chronic multi-symptom illness criteria, of screening out those with conditions tied to mitochondrial impairment—like headache and multiple symptoms—even if symptoms were not acknowledged as persistent over the prior six months. This latter entails some degree of subjectivity—e.g. intermittent headache might not be consistently characterized as “persistent.”

However, it was only by expanding assessment to encompass cases without weakness, and controls that had recent headache and/or recent multiple symptoms, that data were secured to document the importance of such careful selection choices.

Additionally, consistent with our own and others' findings in Gulf War illness studies, homogeneity in sex and ethnicity may reduce variance enhancing power at a given sample size, which may benefit detection of true effects and associations. Additionally, evidence for differences in symptoms, physiology and biomarkers by sex and ethnicity in veterans with GWI also supports assessments of differences as a function of demographic factors^11,18,45–47^. Controls as well as cases may contribute to differences as a function of demographics, as can also be seen in other GWI data^47^. Males and Caucasians predominate among Gulf War veterans and veterans with GWI, so findings in this group are of separate high importance. Extending the sample beyond the dominant demographic group was imperative for understanding these distinctions and affirms that separate analysis may be warranted when other groups are included. Indeed, it is the recognition that distinct demographic groups may differ that leads to the endorsement for including them in studies, but this inclusion can only help those groups if analysis is conducted to identify potential presence of such differences.

Findings in the replication group of veterans with GWI, documenting bioenergetic impairment, match the prior finding with strikingly similar results. They cohere with a report of increased mitochondrial DNA mutations and impaired respiratory chain function in veterans with GWI vs controls, assessed however in peripheral blood mononuclear cells^13^. Findings are reinforced by data in Gulf War animal models documenting alterations in mitochondrial proteomics, gene expression and lipids^14,15,17^; and more relevant to the present study documenting muscle mitochondrial impairment^48^, including in association with muscle atrophy^19^. Most relevantly, they accord with data documenting impaired mitochondrial respiratory chain function from muscle biopsy tissue in veterans with GWI^26^, and a correlation of muscle mitochondrial measures with GWI severity; a correlation to symptoms was not observed for hsCRP (Pearson correlation coefficient), a marker of inflammation^26^. They also comport with recent preliminary reports that mitochondrial haplogroups correlate with severity of GWI^18^. All this is in context that exposures tied by compelling evidence to GWI such as acetylcholinesterase inhibitor exposures (sarin/cyclosarin nerve gas, pesticides including organophosphates and carbamates, and pyridostigmine bromide given as a nerve agent pretreatment adjunct) produce toxicity in substantial part through mitochondrial effects^7–9,12^. This is also in context that double-blind placebo-controlled randomized trial evidence has reported that coenzyme Q10, which bypasses many mitochondrial impairments^49^, significantly reduced symptoms and improved objectively-measured physical function in veterans with GWI^11^. Of note, in referencing “mitochondrial dysfunction” for the present purpose, we encompass impaired energy production by mitochondria from any of a panoply of potential causes, not necessarily exclusively respiratory chain impairment per se. As above, impairment in mitochondrial biogenesis, transport, substrate delivery to mitochondria, and a range of other factors could each in principle contribute to impaired bioenergetics and reduced ATP production in muscle, and consequently, prolonged PCr recovery time constant. That is to say, the findings here support “mitochondrial impairment” construed in the most liberal sense, as reduced ATP production by mitochondria, whatever the cause (motivating use of the term “bioenergetics” in most instances through this manuscript). However, the recent findings documenting respiratory chain impairment in muscle biopsy tissue in veterans with GWI relative to controls add potently to prospects that respiratory chain impairment is the foundation—or at least one foundation—of bioenergetic findings.

The headache findings extending to “healthy” controls, in the context of other evidence tying headaches to mitochondrial and bioenergetic impairment, will be published separately. Briefly, headache is not infrequently tied to mitochondrial impairment specifically^31–33^, or cell energy supply–demand more broadly. Headache is increased with either increased energy demand or reduced energy supply. This is further reflected by effective treatments for headache, including coenzyme Q10^11,50–52^ which increases energy supply, and Botox® which reduces muscle contraction and thereby reduces cell energy demand^53–59^. Headaches in "healthy" controls—i.e. those not meeting criteria for chronic multi-symptom illness—even if not acknowledged (or not acknowledged yet) to be chronic or persistent, may be pointers to bioenergetic impairment.

These findings are clinically relevant in that they affirm that male white VGWI have bioenergetic impairment. They affirm that the extent of bioenergetic impairment assessed by muscle post-exercise phosphocreatine recovery time constant relates to the degree of self-reported muscle weakness, both validating self-reported muscle weakness and underscoring that the utility of the instrument for bioenergetic assessment indeed rests on matching the tissue assessed to an affected tissue. Moreover, further substantiation of a bioenergetic foundation for GWI has implications for treatment, and in fact most reported successful treatments to date—from coenzyme Q10^11^and other supplements that mitigate mitochondrial impairment^16,60–64^, to treatment of sleep apnea^65^—address bioenergetics. Oxygen is a 13-fold multiplier in ATP produced per glucose molecule, so that in individuals with compromised mitochondrial function/energy production, even modest diminutions in oxygen saturation can have a greatly magnified impact.

The replication sample was of modest size. However, significance despite this—signifying the large effect size—the striking concordance with results of the study being replicated, and the potent significance of the relationship in the combined sample (combining the replication sample with the sample it seeks to replicate), p = 0.001, buttressed confidence in the finding. Moreover, triangulating evidence of mitochondrial impairment in GWI and the study finding of the potent relation of PCr-R to muscle weakness within veterans with GWI, further support the validity of the finding^26^.

Although in one sense results could have been strengthened by focusing more of the sample on the case and control features optimized for replication, it is only by broadening beyond the original sample that we can assert and affirm the critical nature of the original choices, and can better inform future such studies.

## Conclusion

Mitochondrial/bioenergetic impairment was affirmed in veterans with GWI, with a replication sample providing strikingly similar findings to the sample it seeks to replicate. Self-rated muscle weakness in veterans with GWI significantly related to prolonged PCr-R. The fact that symptom status in an organ, muscle, related to bioenergetic status in that organ in a mitochondrially-related condition^26^, reinforces the potential importance of matching organ assessed to one that is impaired in mitochondrial studies.

## Data Availability

Data will be made available on request. Participants were advised that their data would be kept confidential, with only group-level data published. Because specific demographic and symptom information could potentially identify individual veterans, we prefer to share the data on an as requested basis, with assurances of confidentiality protections, rather than in a public-use database. Requests for data should be made to Dr. Golomb (bgolomb@ucsd.edu).
